# Canonical Correlation Analysis on the Association Between Sleep Quality and Nutritional Status Among Centenarians in Hainan

**DOI:** 10.3389/fpubh.2020.585207

**Published:** 2020-11-20

**Authors:** Guangdong Liu, Shanshan Yang, Wei Liu, Shengshu Wang, Penggang Tai, Fuyin Kou, Wangping Jia, Ke Han, Miao Liu, Yao He

**Affiliations:** ^1^State Key Laboratory of Kidney Disease, Beijing Key Laboratory of Aging and Geriatrics, The 2nd Clinical Center, Institute of Geriatrics, Chinese People's Liberation Army General Hospital, Beijing, China; ^2^Department of Disease Prevention and Control, The 1st Medical Center, Chinese People's Liberation Army General Hospital, Beijing, China; ^3^Hospital Management Institute, Chinese People's Liberation Army General Hospital, Beijing, China; ^4^Department of Statistics and Epidemiology, Graduate School, Chinese People's Liberation Army General Hospital, Beijing, China

**Keywords:** centenarians, sleep quality (SQ), canonical correlation analysis, Chinese, nutritional status

## Abstract

**Objective:** To analyze the correlation between nutritional status and sleep quality among centenarians.

**Methods:** A total of 1,002 centenarians in Hainan were included in the full sample survey. The Mini Nutritional Assessment-Short Form (MNA-SF) was used for nutritional risk assessment and the Pittsburgh Sleep Quality Index Scale (PSQI) was used for evaluating sleep quality. Canonical correlation analysis was conducted to analyze their correlation.

**Results:** Two statistically significant (*p* < 0.05) canonical coefficients were found, with the first canonical correlation coefficient having a value of 0.247, eigenvalue of 0.065, and contribution rate of 89.0%. The linear combination of nutrition variable V_1_, mainly determined by MNA1 (appetite loss), MNA5 (dementia/depression), and MNA2 (weight loss), indicates an association with sleep quality; the linear combination of sleep quality W_1_, mainly determined by PSQI1 (subjective sleep quality), PSQI7 (daytime dysfunction), and PSQI2 (sleep latency), indicates an association with nutritional status. Appetite loss, dementia/depression, and weight loss have negative correlations with subjective sleep quality and daytime dysfunction.

**Conclusion:** Among centenarians, the relationship between nutritional status and sleep quality is bidirectional. Furthermore, appetite loss, dementia/depression, weight loss, subjective sleep quality, and daytime dysfunction are the main relevant factors.

## Introduction

An aging population is an inevitable consequence of socioeconomic development. The World Health Organization estimates that the world's population over 60 years old will increase from 900 million in 2015 to 2 billion by 2050, and this rate is still accelerating rapidly. In the future, China may take <20 years to adapt to the changes happening in developed countries such as France, in which the proportion of the population over 60 years old has increased from 10 to 20% in the past 150 years (1). For the growing population of older people, successful aging (SA) is the guarantee and cornerstone to achieve healthy social development. According to worldwide research development and trends, SA is used to describe elder people with bio-psycho-social health who not only have normal physical function in their daily lives but also maintain their mental and emotional health (2, 3). Nutritional status and sleep quality are the two cornerstone factors that comprehensively affect the physical and mental health of the elderly. Their poor condition can lead to comprehensive health consequences such as depression, anger, muscle tension, sympathetic excitement, cognitive disorders, neurological disorders, gastrointestinal, and upper respiratory tract diseases, and even cardiovascular and cerebrovascular injury (4–6). However, there are only a limited number of studies focused on nutrition and sleep status in the healthy elderly, especially centenarians. As previously reported, the proportion of sleep disorders in the elderly group is between 17.7 and 22.2% (7, 8), and the risk ratio of malnutrition is between 4.3 and 34.7% (9, 10). A high rate of sleep disorders and malnutrition in the elderly deserves attention and should be observed.

Previous studies have shown that sleep could affect food intake and is associated with gastrointestinal diseases (11), while nutrition could also affect sleep (12). However, most of the studies only focus on children and young people while research on sleep and nutritional status in the elderly population is limited. Whether the relationship between nutrition and sleep status in the elderly population is similar to that in adults under 65 years old or children is still unclear. In addition, since both nutrition and sleep status are multidimensional and intercorrelated with each other, it is difficult to directly evaluate the association between nutritional status and sleep quality. Canonical correlation analysis, a multidimensional technique, could linearly combine multiple factors into separate sub-health and work pressure groups then analyze the correlation between the two groups' variables (13). Therefore, our study aims to analyze the correlation between nutritional status and sleep quality via canonical correlation analysis using a full sample survey of the centenarians in Hainan, and the results might help improve the overall health of the elderly.

## Methods

### Research Objects

According to the list of centenarians provided by the Department of Civil Affairs of Hainan Province from June 2014 to December 2016, a full sample household survey was conducted on all centenarians in 18 cities (counties) in Hainan Province. Excluding the elderly who died, could not be reached, failed the age check, or did not cooperate with the investigator, a total of 1,002 centenarians were surveyed. The inclusion criteria was (1) Permanent residents of Hainan, China; (2) Age ≥100 years and passed the age check; (3) Be capable of consenting and have agreed to participate. The exclusion criteria was (1) Could not be reached; (2) Failed the age check; (3) Did not cooperate with the investigators. More details may be found in the published research protocol (14). The basic information, including questionnaire interviews, physical examinations, and laboratory blood sample tests, was collected through household surveys. Questionnaire and body indicator measurements (height, weight, waist circumference, and blood pressure) were all conducted by systematically trained local nurses who are able to speak the local language and communicate without barriers in Hainan. Questionnaire using a one-to-one household survey, the nurses asked questions in order then the centenarians answered one by one.

### Definition and Evaluation Criteria

The nutritional status of centenarians was evaluated by Mini Nutritional Assessment-Short Form (MNA-SF) (15). The scale consists of a total of six components including appetite loss, weight loss, mobility, stress or acute disease, dementia or depression, and BMI or CC, with a maximum score of 14 points. A score of 12–14 suggests the person is in good nutrition, 8–11 suggests the person is at risk of malnutrition, and a score of 7 or less indicates the person is malnourished. The higher the score of each component and the total score of MNA-SF, the better the nutritional status. The 6 components were labeled MNA~MNA6 in the statistical analysis.

The Pittsburgh Sleep Quality Index Scale (PSQI) consisting of 23 items was used to evaluate the sleep quality of residents by measuring seven domains: subjective sleep quality, sleep latency, sleep duration, habitual sleep efficiency, sleep disturbances, use of sleeping medication, and daytime dysfunction (16). Scoring of the answers is based on a 0–3 scale in each of the domains. The cumulative score of each domain comprises the total score of PSQI (0–21 points). The higher the score of each domain and the total score of PSQI, the worse the sleep quality. The centenarians with scores >7 (scores 8–21) were identified as poor sleep quality. The 7 domains were labeled PSQI1~PSQI7 in the statistical analysis.

Other variables involved in this study were gender, ethnicity (i.e., Han, Li, and other minorities), marital status (i.e., living in marriage, widowed, and others), and educational level (i.e., illiterate, elementary, and junior high school and above) (14). All information was collected through household surveys conducted by systematically trained Hainan local nurses who are able to speak the local language without communication barriers.

### Statistical Analysis

A significance level of 0.05 for the two-sided test was used for each hypothesis testing. Quantitative data were described by means ± standard deviation, and their differences were compared through an independent sample *t*-test. Categorical data were described in *n* (%), and their differences were compared using a chi-square test. Canonical correlation analysis was used to analyze the correlation between nutritional status and sleep status of centenarians. The sign of the standardized coefficient (whether it is positive or negative) indicates the direction in which the raw variable influences the canonical variate. The magnitude of the standardized coefficient represents the magnitude of the variable's influence on the canonical variate. The larger the absolute value, the greater the contribution of the raw variable to the canonical variate; the canonical variate is mainly determined by this raw variable.

The research data were validated by Epidata 3.1 software. The statistical analysis was performed using the SPSS 26.0 software package.

### Ethics

The CHCCS was conducted in accordance with the Declaration of Helsinki and was approved by the Medical Ethics Committee of the Chinese PLA General Hospital (301hn11-206-01). All participants provided written informed consent before joining the study.

## Results

### Characteristics Description

Of the 1,002 centenarians who completed the survey, eight participants with incomplete primary outcome data were removed and a total of 994 people, including 815 females (82.0%) and 179 males (18.0%), with an average age of 102.77 ± 2.75-years-old were included in the study.

The average score of the study sample on the MNA-SF was 9.23 ± 3.06 points. Two hundred and five (20.6%) were malnourished, of which 23 (12.8%) were male and 182 (22.3%) were female. A total of 123 (12.4%) were in good nutrition, of which 30 (16.8%) were male and 93 (11.4%) were female, with a significant gender disparity (*p* = 0.006).

The average PSQI score was 6.79 ± 3.48 points, and 353 (35.5%) had sleep disorders, of which 65 (36.3%) were male and 288 (35.3%) were female. No significant difference was found between the genders (*p* = 0.805).

The relationship between PSQI scores of centenarians with different nutritional status differed by sex, age group, and education level (Table 1, *p* < 0.05). There was a statistical difference among the scores of centenarians with different sleep conditions on the MNA-SF scale (Table 1, *p* < 0.05).

**Table 1:** Nutritional status and sleep quality of centenarians in Hainan.

**Mean + SD**	**Total (*n* = 994)**	**Malnutrition (*n* = 205)**	**At risk of malnutrition (*n* = 666)**	**Normal (*n* = 123)**	***P***	**Normal (*n* = 641)**	**Had sleep problems (*n* = 353)**	***P***
AGE	102.8 ± 2.8	102.9 ± 2.7	102.8 ± 2.8	102.5 ± 2.6	0.500	102.7 ± 2.7	102.9 ± 2.8	0.163
PSQI	6.8 ± 3.5	7.4 ± 3.6	6.7 ± 3.4	6.3 ± 3.4	0.007	4.6 ± 1.5	10.7 ± 2.4	<0.001
MNA-SF	9.2 ± 2.1	6.3 ± 0.9	9.5 ± 1.1	12.5 ± 0.7	<0.001	9.3 ± 2.0	9.0 ± 2.1	0.027
*N* (%)								
Gender					0.006			0.805
Male	179 (18.0%)	23 (11.2%)	126 (18.9%)	30 (24.4%)		114 (17.8%)	65 (18.4%)	
Female	815 (82.0%)	182 (88.8%)	540 (81.1%)	93 (75.6%)		527 (82.2%)	288 (81.6%)	
Age group					0.029			0.201
100–104	788 (79.3%)	157 (76.6%)	522 (78.4%)	109 (88.6%)		519 (81.0%)	269 (76.2%)	
105–109	175 (17.6%)	42 (20.5%)	124 (18.6%)	9 (7.3%)		103 (16.1%)	72 (20.4%)	
≥110	31 (3.1%)	6 (2.9%)	20 (3.0%)	5 (4.1%)		19 (3.0%)	12 (3.4%)	
Ethnic					0.596			0.353
Han	875 (88.0%)	179 (87.3%)	585 (87.8%)	111 (90.2%)		557 (86.9%)	318 (90.1%)	
Li	106 (10.7%)	25 (12.2%)	70 (10.5%)	11 (8.9%)		75 (11.7%)	31 (8.8%)	
Others	13 (1.3%)	1 (0.5%)	11 (1.7%)	1 (0.8%)		9 (1.4%)	4 (1.1%)	
Marital status					0.642			0.542
Married	98 (9.9%)	17 (8.3%)	67 (10.1%)	14 (11.4%)		68 (10.6%)	30 (8.5%)	
Widowed	832 (83.7%)	172 (83.9%)	556 (83.5%)	104 (84.6%)		531 (82.8%)	301 (85.3%)	
Divorced or never married	64 (6.4%)	16 (7.8%)	43 (6.5%)	5 (4.1%)		42 (6.6%)	22 (6.2%)	
Education level					0.012			0.093
Illiterate	909 (91.4%)	193 (94.1%)	612 (91.9%)	104 (84.6%)		577 (90.0%)	332 (94.1%)	
Primary school	66 (6.6%)	11 (5.4%)	43 (6.5%)	12 (9.8%)		50 (7.8%)	16 (4.5%)	
Middle school or higher	19 (1.9%)	1 (0.5%)	11 (1.7%)	7 (5.7%)		14 (2.2%)	5 (1.4%)	

*PSQI, Pittsburgh sleep quality index; MNA-SF, Mini nutritional assessment short-form*.

### Canonical Correlation Analysis of Nutritional Status and Sleep Quality

#### Spearman's Correlation

The correlation coefficient between the total scores of MNA-SF and PSQI was −0.090 (*p* = 0.004). Table 2 shows the correlation coefficient matrix among the six components of the MNA-SF scale and the seven domains of the PSQI among the centenarians in Hainan. The correlation coefficients among the components are relatively small, with the largest absolute value of −0.191 (*p* < 0.001) being the correlation coefficient between MNA1 (appetite loss) and PSQI1 (subjective sleep quality).

**Table 2:** Spearman's correlation between MNA-SF and PSQI in centenarians.

		**Subjective sleep quality**	**Sleep latency**	**Sleep duration**	**Habitual sleep efficiency**	**Sleep disturbances**	**Use of sleeping medication**	**Daytime dysfunction**
Appetite loss	Spearman's correlation coefficient	**−0.191**	−0.031	**−0.085**	−0.057	**−0.069**	−0.019	**−0.121**
	*P*	0.000	0.332	0.007	0.073	0.029	0.549	0.000
Weight loss	Spearman's correlation coefficient	**−0.095**	0.044	−0.013	−0.050	−0.039	0.025	**−0.085**
	*P*	0.003	0.161	0.681	0.112	0.218	0.422	0.007
Mobility	Spearman's correlation coefficient	**−0.074**	−0.014	0.012	0.035	−0.061	−0.038	−0.025
	*P*	0.019	0.652	0.696	0.275	0.054	0.237	0.438
Stress/acute disease	Spearman's correlation coefficient	−0.015	−0.010	−0.006	0.001	**−0.101**	0.023	0.060
	*P*	0.631	0.757	0.847	0.979	0.001	0.471	0.057
Dementia/depression	Spearman's correlation coefficient	**−0.134**	**−0.083**	−0.027	−0.039	−0.021	0.028	−0.054
	*P*	0.000	0.009	0.404	0.219	0.514	0.384	0.089
BMI	Spearman's correlation coefficient	−0.018	0.019	−0.016	−0.018	−0.041	−0.018	0.019
	*P*	0.567	0.539	0.624	0.568	0.196	0.564	0.548

*Bold value: P <0.05*.

#### Canonical Correlation

The canonical correlation analysis was performed between nutritional variables and sleep quality variables, and the outcome contains 6 pairs of canonical variables. The correlation coefficients of the first two pairs of canonical variates were statistically significant (*p* < 0.05), with the first correlation coefficient having a value of 0.247, an eigenvalue of 0.065, a contribution rate of 89.0%, and a cumulative contribution rate of 89.0%, which was also statistically significant based on the likelihood ratio test (*p* < 0.001). The cumulative contribution rate of the first pair of canonical variates was as high as 89.0%. Thus, only the first pair of canonical variables was analyzed further (Table 3).

**Table 3:** Outcomes of canonical correlation analysis and likelihood ratio test.

	**Canonical correlation**	**λ**	**Proportion**	**Cumulative**	***F***	***P***
1	0.247	0.065	0.890	0.890	2.748	0.000
2	0.152	0.024	0.058	0.948	1.755	0.007
3	0.125	0.016	0.022	0.971	1.474	0.080
4	0.097	0.009	0.016	0.986	1.149	0.315
5	0.062	0.004	0.009	0.995	0.748	0.611
6	0.026	0.001	0.004	0.999		

The canonical correlation analysis was repeated in different gender groups. The canonical correlation coefficient of the first pair of canonical variates in men was 0.412, with an eigenvalue of 0.204 and a contribution rate of 65.7% (*p*-value of 0.003 from likelihood ratio test). Three pairs of canonical variates in the female centenarians were statistically significant. The canonical correlation coefficient of the first pair of canonical variates was 0.239, its eigenvalue was 0.060, and its contribution rate was 87.8% (*p* < 0.001 from likelihood ratio test) (Table 4). Since the cumulative contribution rate of the first pair of canonical variates in both males and females is relatively large, only the first pair of canonical variates were analyzed further.

**Table 4:** Outcome of canonical correlation analysis and likelihood ratio test in males and females.

**Gender**	**Canonical correlation**	**λ**	**Proportion**	**Cumulative**	***F***	***P***
Male						
1	0.412	0.204	0.657	0.657	1.746	0.003
2	0.335	0.126	0.134	0.791	1.347	0.104
3	0.254	0.069	0.100	0.891	0.989	0.473
4	0.205	0.044	0.061	0.952	0.694	0.757
5	0.077	0.006	0.042	0.994	0.169	0.985
6	0.009	0.000	0.006	1.000		
Female						
1	0.239	0.060	0.878	0.878	2.511	0.000
2	0.173	0.031	0.053	0.932	1.916	0.002
3	0.151	0.023	0.029	0.960	1.638	0.037
4	0.105	0.011	0.023	0.983	1.162	0.305
5	0.075	0.006	0.011	0.994	0.844	0.536
6	0.024	0.001	0.006	0.999		

**Figure 1:**
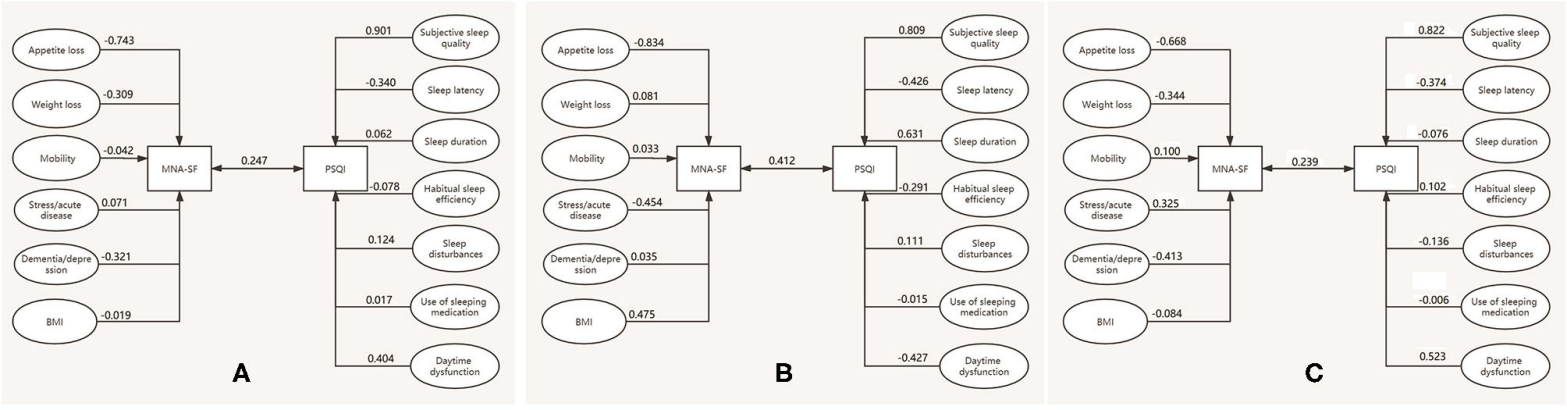
Canonical correlation structure chart of MNA-SF and PSQI. **(A)** The whole sample, **(B)** male centenarians, **(C)** female centenarians.

V_1_ represented the first pair of canonical variates for nutritional status: V_1_ = −0.743MNA1 – 0.309MNA2 – 0.042MNA3 + 0.071MNA4 – 0.321MNA5 – 0.019MNA6; W_1_ represented the first pair of canonical variates for sleep quality: W_1_ = 0.901PSQI1 – 0.340PSQI2 + 0.062 PSQI3 – 0.078 PSQI4 + 0.124 PSQI5 + 0.017 PSQI6 + 0.404 PSQI7 (Figure 1A). The linear combination of nutrition variable V_1_, mainly determined by MNA1 (appetite loss), MNA5 (dementia/depression), and MNA2 (weight loss) indicates an association with sleep quality; the linear combination of sleep quality W_1_, mainly determined by PSQI1 (subjective sleep quality), PSQI7 (daytime dysfunction), and PSQI2 (sleep latency) indicates an association with nutritional status.

In the male centenarians:

V_1_ = −0.834MNA1 + 0.081MNA2 + 0.033MNA3 – 0.454MNA4 – 0.035MNA5 – 0.475MNA6; wl = 0.809PSQI1 – 0.426PSQI2 + 0.631 PSQI3 – 0.291 PSQI4 + 0.111 PSQI5 – 0.015 PSQI6 – 0.427 PSQI7 (Figure 1B). The linear combination of the first canonical variate V_1_ for nutritional status is mainly determined by MNA1 (appetite loss), MNA4 (stress/acute disease), and MNA6 (BMI). The linear combination of the first canonical variate W_1_ for sleep quality is mainly determined by PSQI1 (subjective sleep quality), PSQI3 (sleep duration), PSQI7 (daytime dysfunction), and PSQI2 (sleep latency).

In female centenarians:

V_1_ = −0.668MNA1 – 0.344MNA2 + 0.100MNA3 + 0.325MNA4 – 0.413MNA5 – 0.084MNA6; wl = 0.822PSQI1 – 0.374PSQI2 – 0.076 PSQI3 + 0.102PSQI4 – 0.136 PSQI5 – 0.006 PSQI6 + 0.523 PSQI7 (Figure 1C). The linear combination of the first canonical variate V_1_ for nutritional status is mainly determined by MNA1 (appetite loss), MNA5 (dementia/depression), and MNA2 (weight loss). The linear combination of the first canonical variate W_1_ for sleep quality is mainly determined by PSQI1 (subjective sleep quality), PSQI7 (daytime dysfunction), and PSQI2 (sleep latency).

## Discussion

This study found that the proportion of participants with malnutrition was 20.6%, of which 12.8% were male, and 22.3% were female, with significant gender disparity. On the other hand, the proportion of participants with sleep disorders was 35.5%, 36.3% for males and 35.3% for females, with no statistically significant gender disparity. There was a positive correlation between nutritional status and sleep quality in the elderly population, that is, the higher the score on the MNA-SF scale (i.e., good nutritional status), the lower the PSQI score (i.e., good sleep quality). The first pair of canonical correlation coefficients between the nutritional status and sleep status had a value of 0.247 and the contribution rate was 89.0%. The main components for nutritional status were appetite loss, dementia/depression, and weight loss. The main components for sleep quality were subjective sleep quality, daytime dysfunction, sleep latency, and sleep quality. There was significant gender disparity in terms of the canonical correlation coefficients.

A survey based on long-lived elderly (≥90 years old) in Dujiangyan, China showed that the prevalence of sleep disorders is 22.2% (8). A Taiwanese survey based on the elderly over 65 years old showed that the prevalence of poor sleep was 6% (7). The results of this study showed that the prevalence of poor sleep quality in the elderly in Hainan is 35.5%. This difference may be related to the age of the included population. It also suggests that as age increases, the sleep quality is at risk of worsening. This is consistent with the findings of a previous meta-analysis by Ohayon et al. (17) that sleep quality of all age groups, especially those after 60 years of age, will deteriorate with age.

According to a review of nutritional status survey measured by MNA-SF, the prevalence of the risk of malnutrition (score ≤ 11 points) was 8–76% among elderly in the community (18). The Japanese Centenarian Nutrition Survey showed that the proportion of malnutrition risk was 34.7% (10), while the proportion of malnutrition risk in our sample reached 87.6%, which may be related to the regional dietary differences (19, 20). The risk of having such a high proportion of malnutrition among centenarians should be taken into account, given that a number of previous studies have shown that malnutrition in the elderly is associated with greater risks of acquiring consciousness disorders (21), depression (9), lower quality of life (22), and a higher 5-year mortality rate (23).

Previous sleep and nutrition studies have mostly focused on the effects of sleep on dietary intake and obesity in adolescents or adults (11). The results showed that adolescents or adults with sleep problems are more likely to be obese and tend to consume more energy (11, 24). As these studies paid more attention to the relationship between sleep duration and obesity (BMI, waist circumference); there was very limited research on comprehensive sleep quality and different components of nutritional status.

In this study, we found a positive correlation between nutritional status and sleep quality among the centenarians, that is, centenarians with better sleep quality have a better nutritional status, which is dissimilar to findings from studies based on adolescent populations. The inconsistent results may be related to the differences between the elderly and the young in terms of chronic physiological changes (25), muscle fat percentage, and metabolic levels during sleep time (26, 27).

At the same time, this study also found a strong correlation between nutrition variables, such as appetite loss, dementia/depression, and weight loss, and sleep status variables, which included subjective sleep quality, daytime dysfunction, sleep latency, in female centenarians. In contrast, other factors, such as sleep duration and dementia or depression had a stronger correlation in male centenarians. Furthermore, weight loss, which was the main aspect in the female centenarians, was replaced by stress/acute disease and BMI in male centenarians. These findings suggest that the improvement of sleep quality and nutritional status of elderly people could be started from different directions in males and females. For example, if an elderly male has a sleep disorder concerning sleep duration, he may consider improving the quality of sleep by improving his nutritional status by enhancing food diversity, boosting appetite, reducing stress, and improving acute illness, etc. On the other hand, if an elderly female has a sleep disorder concerning sleep duration, improving her nutritional status may not be as effective in improving her quality of sleep. And if an elderly female wants to improve her subjective sleep quality, improving her nutritional status by boosting appetite and elevating mood may be an effective way.

This study has certain limitations. First, the population in the study is a large sample of centenarians in an island environment, and therefore the conclusion needs to be generalized with caution. Second, the number of male centenarians in this study was considerable less than that of female centenarians, therefore the results of its association analysis might be biased.

### Conclusions

Despite these limitations, this study provided an in-depth analysis of the relationship between nutritional status and sleep quality of centenarians in Hainan. It was found that malnutrition and sleep disorder are prevalent among the centenarians, and positively correlated with one another. Furthermore, this study shows the relationship between nutritional status and sleep quality is bidirectional. Based on these findings, specific aspects of sleep and nutrition problems in the elderly population could be analyzed and targeted preventive interventions could be strengthened to promote the overall health of the elderly and achieve healthy aging.

## Data Availability Statement

The dataset used in this study can be obtained from the corresponding authors by a reasonable requests.

## Ethics Statement

The studies involving human participants were reviewed and approved by the CHCCS was conducted in accordance with the Declaration of Helsinki and was approved by the Medical Ethics Committee of the Chinese PLA General Hospital (301hn11-206-01). The ethics committee waived the requirement of written informed consent for participation.

## Author Contributions

GL, SY, WL, ML, and YH contributed to data analysis and manuscript writing. SY, SW, PT, FK, WL, WJ, KH, ML, and YH contributed to study design and data collection. All authors contributed to manuscript revision and approval of final submission.

## Conflict of Interest

The authors declare that the research was conducted in the absence of any commercial or financial relationships that could be construed as a potential conflict of interest.
